# A Coupled Visual and Inertial Measurement Units Method for Locating and Mapping in Coal Mine Tunnel

**DOI:** 10.3390/s22197437

**Published:** 2022-09-30

**Authors:** Daixian Zhu, Kangkang Ji, Dong Wu, Shulin Liu

**Affiliations:** 1 College of Communication and Information Engineering, Xi’an University of Science and Technology, Xi’an 710054, China; 2 College of Electrical and Control Engineering, Xi’an University of Science and Technology, Xi’an 710054, China

**Keywords:** visual feature tracking, sensor fusion, SLAM, tightly coupled

## Abstract

Mobile robots moving fast or in scenes with poor lighting conditions often cause the loss of visual feature tracking. In coal mine tunnels, the ground is often bumpy and the lighting is uneven. During the movement of the mobile robot in this scene, there will be violent bumps. The localization technology through visual features is greatly affected by the illumination and the speed of the camera movement. To solve the localization and mapping problem in an environment similar to underground coal mine tunnels, we improve a localization and mapping algorithm based on a monocular camera and an Inertial Measurement Unit (IMU). A feature-matching method that combines point and line features is designed to improve the robustness of the algorithm in the presence of degraded scene structure and insufficient illumination. The tightly coupled method is used to establish visual feature constraints and IMU pre-integration constraints. A keyframe nonlinear optimization algorithm based on sliding windows is used to accomplish state estimation. Extensive simulations and practical environment verification show that the improved simultaneous localization and mapping (SLAM) system with a monocular camera and IMU fusion can achieve accurate autonomous localization and map construction in scenes with insufficient light such as coal mine tunnels.

## 1. Introduction

Simultaneous localization and mapping (SLAM) is an important technology for mobile robots to explore unknown areas [1]. The mainstream SLAM methods are divided into two types: vision and laser. A series of efficient, real-time pose-estimation methods have been proposed over the years. Laser SLAM is relatively mature in terms of theory, framework, and technology, and has matured applications in many aspects [2]. For example, the KUKA Navigation Solution in 2D-laser SLAM is used in sweeping robots. However, 2D SLAM can only obtain single plane information in the environment, which is not suitable for application in large complex scenes. In order to obtain more abundant environmental information, multi-line (Light Detection And Ranging) LiDAR or vision is usually used for positioning and mapping [3]. The increase in the number of radar lines means that more information can be obtained. At the same time, it also represents an increase in hardware costs. Vision sensors are cheaper compared to LiDAR. Maps constructed using vision sensors contain more information. Low power consumption and low weight make it possible to deploy vision sensors on mobile platforms with limited load. Vision SLAM has a wide range of applications both indoors and outdoors [4]. The image information obtained from the vision sensors is used to calculate the camera motion between adjacent images and thus estimate the camera motion trajectory. This method is called inter-frame estimation. As an important part of vision SLAM, the accuracy of inter-frame matching directly affects the results of localization and map building.

In recent years, many excellent visual SLAM algorithms have been proposed, and the real-time performance and positioning accuracy have been greatly improved. The method of the characteristic point is most commonly used for inter-frame estimation. Feature points such as corner points, edge points, and blocks in adjacent images are extracted and used to estimate camera motion. Feature point-extraction algorithms include Scale Invariant Feature Transform (SIFT) [5], Speeded Up Robust Features (SURF) [6], Harris [7], Oriented Fast and Rotated Brief (ORB) [8], etc. In these feature-extraction methods, only the significant feature points in the image are extracted. Also to improve the real-time performance of the algorithm, the distribution of feature points is sparse. This makes the visual SLAM more sensitive to illumination and features are often lost under drastic changes in illumination and fast camera motion. For example, both ORB-SLAM2, proposed by Raul Mur-Artal et al., and DSO (Direct Sparse Odometry), proposed by Jakob et al. [9,10], localize by visual methods. They are able to achieve good localization accuracy when the ambient lighting conditions are good. However, they tend to perform poorly under fast motion and poor illumination conditions.

With the increasing requirements for localization accuracy and robustness in different application scenarios, it is not enough to collect information from a single sensor and then calculate the position. Therefore, more and more researchers are focusing on multi-source fusion approaches [11]. There are various ways of using multi-source fusion. Among them, the fusion of sensors is the most commonly used [12]. This includes fusion applications of two or more sensors in cameras, LiDAR, Inertial Measurement Unit (IMU), and GPS. There is also multi-feature fusion, which includes the extraction of features such as points, lines, and grayscale values to obtain multiple feature map elements.

Monocular visual SLAM can collect enough environmental information through cameras in indoor scenes with good lighting conditions and rich features. Then the camera state is estimated and the positional pose is calculated. However, when the camera moves rapidly, it is easy to lose point features with monocular visual SLAM [13]. Because of the limitations of camera frame rate and resolution, it is difficult to achieve accurate localization in fast motion. With its high acceleration and angular velocity measurement rates, the IMU can be used to assist vision sensors for feature tracking. Although the bias and noise of the IMU cause an accumulation of errors and drifts, they can be corrected by vision methods. The use of different sensors can complement each other to provide more accurate global or local positioning measurements for mobile robots. Many excellent SLAM algorithms for sensor fusion have been proposed in recent years. Methods that fuse vision and IMU, such as ORB-SLAM3 [14], use long-term data correlation to achieve high-precision localization in large scenes. Its fast and accurate IMU initialization and multi-session map merging capabilities enable robust operation in real-time in all kinds of scenes, but this comes at a higher computational cost. VI-DSO [15], proposed by Lukas et al., also balances speed and accuracy and enables real-time centimeter-level localization. However, this method requires a longer time for IMU initialization, even more than 30 s under poor lighting conditions. Based on the extended Kalman filter, the MSCKF [16] proposed by Mourikis et al. is a visual-inertial odometer that fuses visual and inertial information. Instead of estimating feature point states as system states, it uses a sliding window strategy to combine features from multiple observation frames. The filter is updated using the pose constraint between observation frames, which effectively improves the real-time performance of the algorithm. However, it has more error and insufficient localization accuracy when applied in large scenes without loopback detection. Leutenegger et al. proposed a nonlinear optimization-based visual-inertial navigation fusion method, OKVIS [17]. The scheme supports binocular cameras and uses a sliding window method to construct a minimum error function by visual reprojection constraints and IMU measurement constraints. OKVIS uses the First-Estimates Jacobian (FEJ) to ensure the consistency of the system. However, its slow computation speed is not suitable for scenes that require real-time feedback such as autonomous mobile robots. The VINS-Mono [18] proposed by Shen Shaojie et al. uses an optical flow method for motion tracking and pre-integrates IMU data.

Its fast initialization process and visual-inertial tightly coupled nonlinear optimized estimator achieve accurate pose estimation of the unmanned aircraft. Since the front-end uses the optical flow method for tracking matching without extracting feature descriptors, feature points will be lost when the frame blurs due to violent motion. Moreover, it is more sensitive to light changes, which affects the positioning accuracy.

Because there are some limitations in all of the above methods, this paper proposes a localization and mapping method based on VINS-Mono. It achieves high accuracy and real-time mobile robot trajectory estimation and map construction through the coupling of vision and IMU. The method combines ORB features and line features to improve the accuracy of robot feature extraction in scenes with poor lighting conditions such as rugged ground and coal mine tunnels. It also reduces the feature loss due to motion blur and illumination changes. In addition, the method uses IMU data to assist visual features for localization and tracking, which provides more constraints on the estimation of the system state and improves the system localization accuracy. We experimented with the proposed method in real scenarios and EuRoC datasets [19]. The experimental results of the real Tunnel environment and the EuRoC dataset show that the improved algorithm can track stably in environments with fast motion and poor lighting conditions, and obtain higher accuracy of the positional estimation results.

The chapters of this article are organized as follows: Section 2 presents our work for the tightly coupled approach for the visual-inertial SLAM algorithm. The relevant experiments and results are analyzed in Section 3, followed by conclusions in Section 4.

## 2. Materials and Methods

**Figure 1:**
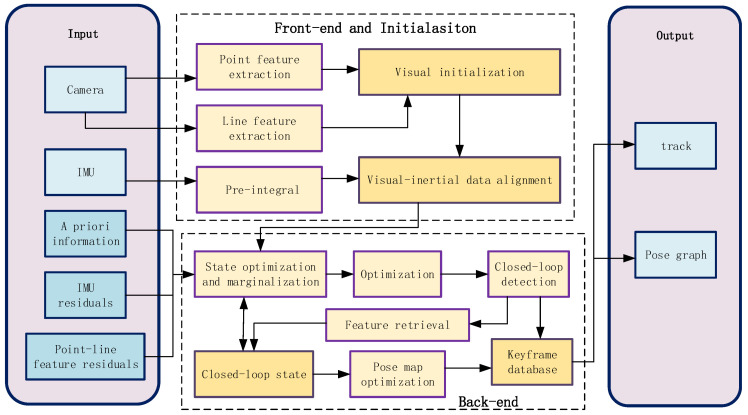
System block diagram.

The system block diagram of the positioning and mapping algorithm based on the fusion of visual features and IMU proposed is shown in Figure 1. The positioning and mapping algorithm for the fusion of visual features and IMU is mainly divided into front-end, system initialization, and back-end.

### 2.1. Front-End Data Association

In visual SLAM, the front-end processing is to extract the feature points of adjacent frames from the image information collected by the camera, and then perform feature-point matching to calculate the correlation information between image frames. The pre-integration of the IMU information is used as a constraint between image frames [20]. The back-end recovers the depth information from the input-associated information and generates a pose graph. Feature maps are constructed with feature points for closed-loop detection and subsequent robot repositioning. In SLAM, the front-end processing requires not only the accuracy of feature-point matching but also real-time calculation. By comparing the advantages and disadvantages of various feature-point extraction methods, ORB features are finally selected as the feature-extraction object of this system to meet the accuracy and real-time performance of feature extraction and matching [21]. In addition, compared with point features, line features provide more information about the geometric structure of the environment, and have better robustness to illumination changes and texture sparsity.

#### 2.1.1. Extraction and Matching of Image Feature Points

The ORB feature is a highly matching computationally efficient feature that makes the features from accelerated segment test (FAST) directional and uses the binary descriptor BRIEF to describe them [22,23,24].

The FAST feature points do not have rotation invariance, so it is necessary to add a direction description to each feature point by the intensity centroid method. The implementation steps of the algorithm are as follows. First, the moment of the image block is defined in the image block as mentioned in [22], and shown in the following formula:

(1) $$m_{pq}=\sum_{x,y\in B}x^{p}y^{q}I\left(x,y\right)\;\;p,q\in \left\{0,1\right\}$$

where *I*(*x*, *y*) is the gray value of the pixel. The centroid is expressed as:

(2) $$C=\left(\frac{m_{10}}{m_{00}},\frac{m_{01}}{m_{00}}\right)$$

the direction of the feature point is the line connecting the geometric center O and the centroid C of the image block, and the direction vector is $\vec{\mathrm{OC}}$.

The FAST feature points have scale invariance and rotation invariance through the image pyramid and gray centroid method. BRIEF is a binary descriptor and uses Hamming distance to do matching on feature points. The XOR operation greatly improves the computational efficiency and makes the matching of ORB features have an inherently real-time capability.

#### 2.1.2. Extraction and Matching of Image Feature Lines

The extraction of feature lines is based on the Line Segment Detector (LSD) [25] straight-line detection algorithm. This achieves sub-pixel accuracy of line segment detection results and controls the number of false detections.

The LSD algorithm uses the image pyramid method to perform Gaussian blurring on the original image to construct a scale space. The direction in which the image pixels change rapidly is the image-gradient direction, and the contour-line direction is perpendicular to the gradient direction. The area composed of pixels with approximately the same gradient direction in the image of the same scale is called the line-support region.

The description of the line segment adopts the LBD [26] descriptor, which defines *dL* as the line-segment direction, and $d_{⊥}$ as the line-segment orthogonal direction. The construct strip descriptor *BD*_*j*_ is computed from the strips *B*_*j*_, *B*_*j*−1_, and *B*_*j*+1_ in the line support domain.

The expression for the *k* row of the strip is:

(3) $$\{\begin{array}{c} v1_{j}^{k}=\lambda \sum_{gd_{⊥}>0}gd_{⊥}\;\;v2_{j}^{k}=\lambda \sum_{gd_{⊥}<0}gd_{⊥}\\ v3_{j}^{k}=\lambda \sum_{gd_{L}>0}gd_{⊥}\;\;v4_{j}^{k}=\lambda \sum_{gd_{L}<0}gd_{⊥} \end{array}$$

(4) $$\lambda =f_{g}\left(k\right)f_{l}\left(k\right)$$

where *g* represents the image pixel gradient value. $gd_{⊥}$ and $gd_{L}$ represent the projection of gradient values in two directions. λ is the Gaussian coefficient; *fg*(*k*) is the global weighted Gaussian function, which reduces the influence of the gradient of pixels farther from the edge of the line segment on the descriptor; *fl*(*k*) is the local weighted Gaussian function to reduce the boundary effect which comes from the descriptor transitions from one region to another.

The LBD descriptor matrix corresponding to each *BD*_*j*_ is:

(5) $$BDM_{j}=\left[\begin{array}{ccc} v1_{j}^{1} & \cdots & v1_{j}^{n}\\ \vdots & \ddots & \vdots \\ v4_{j}^{k} & \cdots & v4_{j}^{n} \end{array}\right]$$

*BD*_*j*_ consists of the mean direction *M*_*j*_ of *BDM*_*j*_ and the standard deviation vector *S*_*j*_:

(6) $$\mathrm{LBD}={\left(M_{1}^{T},S_{1}^{T},\ldots ,M_{m}^{T},S_{m}^{T}\right)}^{T}$$

Line-feature matching is achieved by judging the Hamming distance of the LBD descriptor. The matching results with too short line segments and too large included angles are eliminated.

#### 2.1.3. IMU Pre-Integration

**Figure 2:**
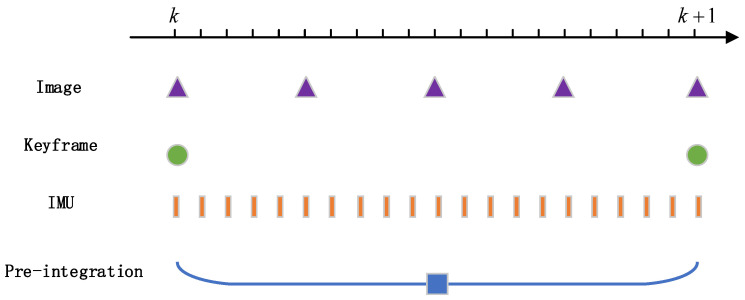
IMU pre-integration.

In general, the sampling frequency of the camera is 30 Hz, whereas the sampling frequency of the IMU can reach up to 1000 Hz. The keyframe mechanism is often used to guarantee the real-time nature of SLAM. As shown in Figure 2, there are many IMU data between adjacent keyframes, and the motion information between keyframes can be calculated by the method of multi-view geometry [27]. The IMU integral term in the world coordinate system contains the rotation matrix *Rbwk* of the IMU coordinate system relative to the world coordinate system. During the optimization process, the change of the key frame bit pose causes the corresponding *Rbwk* to change as well. Then it is necessary to repeat the integration, leading to a large increase in computation. The pre-integration means that the reference coordinate system for IMU integration is converted to the body coordinate system of the previous frame, then the motion between the two frames is calculated to avoid double integration.

The moments *t*_*k*_ and *t*_*k*__+1_ correspond to two consecutive keyframes *x*_*k*_ and *x*_*k*__+1_. The position $p_{b_{k}}^{w}$, velocity $v_{b_{k}}^{w}$, and rotation state $q_{b_{k}}^{w}$ of the system are propagated based on IMU measurements in the interval [*t*_*k*_,*t*_*k*__+1_]. *b*_*k*_ represents the IMU body coordinate system at time *k*, and *w* represents the world coordinate system.

(7) $$\{\begin{array}{c} p_{b_{k+1}}^{w}=p_{b_{k}}^{w}+v_{b_{k}}^{w}∆t_{k}+\iint_{t\epsilon \left[t_{k},t_{k+1}\right]}\left(R_{t}^{w}\left(a_{t}-b_{a_{t}}-n_{a}\right)-g^{w}\right)dt^{2}\\ v_{b_{k+1}}^{w}=v_{b_{k}}^{w}+\int_{t\epsilon \left[t_{k},t_{k+1}\right]}(R_{t}^{w}\left(a_{t}-b_{a_{t}}-n_{a}\right)-g^{w})dt\\ q_{b_{k+1}}^{w}=q_{b_{k}}^{w}⊗\int_{t\epsilon \left[t_{k},t_{k+1}\right]}\frac{1}{2}q_{t}^{b_{k}}⊗\left(w_{t}-b_{w_{t}}-n_{w}\right)dt \end{array}$$

The propagation of states such as system position, velocity, and rotation depends on the position $p_{b_{k}}^{w}$, velocity $v_{b_{k}}^{w}$**,** and rotation $q_{b_{k}}^{w}$ at keyframe *x*_*k*_ moments. When these initial states change after optimization, the state propagation needs to be repeated. This will waste a lot of computing resources. The left side of Equation (8) should be multiplied by $R_{b_{k}}^{w}$, and the reference coordinate system from the world coordinate system adjusted to the body coordinate system of the keyframe at *k* time. This will achieve the relative motion delta independent of the state of *x*_*k*_:

(8) $$\{\begin{array}{c} R_{w}^{b_{k}}p_{b_{k+1}}^{w}=R_{w}^{b_{k}}(p_{b_{k}}^{w}+v_{b_{k}}^{w}∆t_{k}-\frac{1}{2}g^{w}∆t_{k}^{2})+\alpha_{b_{k+1}}^{b_{k}}\\ R_{w}^{b_{k}}v_{b_{k+1}}^{w}=R_{w}^{b_{k}}(v_{b_{k}}^{w}-g^{w}∆t_{k})+\beta_{b_{k+1}}^{b_{k}}\;\;\\ q_{w}^{b_{k}}⊗q_{b_{k+1}}^{w}=\gamma_{b_{k+1}}^{b_{k}} \end{array}$$

(9) $$\{\begin{array}{c} \alpha_{b_{k+1}}^{b_{k}}=\iint_{t\epsilon \left[t_{k},t_{k+1}\right]}\left(R_{t}^{b_{k}}\left(a_{t}-b_{a_{t}}-n_{a}\right)\right)dt^{2}\\ \beta_{b_{k+1}}^{b_{k}}=\int_{t\epsilon \left[t_{k},t_{k+1}\right]}(R_{t}^{w}\left(a_{t}-b_{a_{t}}-n_{a}\right)-g^{w})dt\\ \gamma_{b_{k+1}}^{b_{k}}=\int_{t\epsilon \left[t_{k},t_{k+1}\right]}\frac{1}{2}\Omega \left(w_{t}-b_{w_{t}}-n_{w}\right)\gamma_{t}^{b_{k}}dt \end{array}$$

The reference coordinate system in the integral term is the body coordinate system of the frame *k*. The integral term for the frame *k* + 1 is only related to ***a*_*t*_** and ***w*_*t*_** at different moments. Even if the state of the key frames, such as position, velocity, and rotation is adjusted during optimization, it will not have any effect on the integral term and avoids repeated integration.

### 2.2. System Initialization

**Figure 3:**
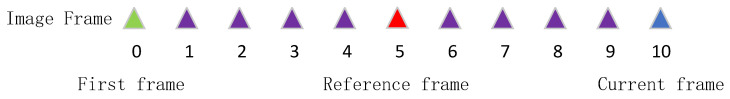
Image frame initialization.

By extracting and matching point and line features, the association between pixels of adjacent frames can be established. Through visual initialization, the pose in the three-dimensional space of the continuous frame camera and the landmark composed of points and lines can be obtained. Assuming that the image frame to be initialized in the sliding window is shown in Figure 3, the goal of visual initialization is to calculate the landmark and the pose of the camera in the sliding window through the structure from motion (SFM) [28].

The reference frame is selected by taking the keyframe with more feature matches in the sliding window as the reference frame. The reference frame and the current frame are polar-constrained and triangulated to calculate the bit pose from the current frame to the reference frame and the 3D roadmap of the co-view. For a frame between the reference frame and the current frame, Perspective-n-points (PnP) [14] can be used to calculate the bit pose of that frame to the current frame, then to triangulate the waymark points of that frame to the current frame. The same operation is performed for a frame between the first frame and the reference frame. Finally, the other unrecovered waypoints are trigonometric again, and all the bit-postures and waypoints in the obtained sliding window are optimized to complete the visual initialization.

Finally, the trajectories calculated by the vision and the IMU are aligned. Then the gyroscope bias, initial velocity, gravity, and scale factor can be assign at the start moment. This completes the system initialization process.

### 2.3. The Back-End Fusion Method

Through the extraction and matching of point-line features and visual initialization, pure vision can resolve adjacent frame poses and co-visualized 3D landmarks. The reprojection residuals are established as the association between image frames for optimizing the poses. The IMU data are pre-integrated as the key inter-frame constraint and also used to establish the image inter-frame constraint [29]. Finally, the optimization based on sliding windows is performed to estimate the optimal poses.

#### 2.3.1. Visual Feature Point and Feature Line Reprojection Model

The visual feature point reprojection residual describes the distance between the projection point and the observation point of the landmark in the three-dimensional space under the normalized camera coordinate system.

**Figure 4:**
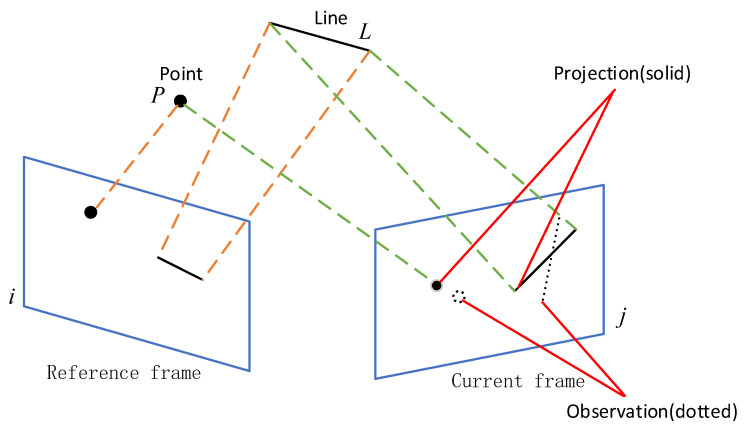
Reprojection error of point and line features.

As shown in Figure 4, the visual observations of map point *P* on the plane of reference frame *i* are converted to the pixel coordinates of the current *j* camera coordinate system, and the defined reprojection residual term is:

(10) $$r_{f}\left({\hat{z}}_{f_{k}}^{C_{i}},\chi \right)=\left[\begin{array}{c} \begin{array}{c} \frac{x^{c_{j}}}{z^{c_{j}}} \end{array}-u_{f_{k}}^{c_{j}}\\ \begin{array}{c} \frac{y^{c_{j}}}{z^{c_{j}}} \end{array}-v_{f_{k}}^{c_{j}} \end{array}\right]$$

(11) $$\left[\begin{array}{c} x^{c_{j}}\\ y^{c_{j}}\\ z^{c_{j}} \end{array}\right]=T_{b}^{c}T_{w}^{b_{j}}T_{b_{i}}^{w}T_{c}^{b}\frac{1}{\lambda_{k}}\left[\begin{array}{c} u_{f_{k}}^{c_{i}}\\ v_{f_{k}}^{c_{i}}\\ 1 \end{array}\right]$$

The reprojection error of the feature line describes the distance between the projected position of the two endpoints of the line on the normalized image coordinate system and the predicted position obtained by inter-frame transformation. The reprojection error can be expressed as:

(12) $$r_{L}\left({\hat{z}}_{l}^{C_{j}},\chi \right)=\left[\begin{array}{c} d_{1}\\ d_{2} \end{array}\right]=\left[\begin{array}{c} \frac{p_{s}^{‘T}l^{‘}}{\sqrt{l_{1}^{2}+l_{2}^{2}}}\\ \frac{p_{e}^{‘T}l^{‘}}{\sqrt{l_{1}^{2}+l_{2}^{2}}} \end{array}\right]$$

where *L* is a straight line in space. Its projected position is expressed as *l* = [*l*_1_, *l*_2_, *l*_3_], and its endpoints are *ps* and *pe*. The predicted position *l*‘, endpoints *ps*‘ and *pe*‘ are obtained by inter-frame transformation.

According to the visual point reprojection formula and the line reprojection formula, the Jacobian matrix relative to each optimization variable is obtained for back-end optimization.

#### 2.3.2. IMU Pre-Integration Residual Model

The IMU pre-integration residual describes the difference between the measured value calculated by IMU pre-integration and the estimated value calculated by pose estimation. The optimized object is the pose of the keyframes. Assuming that the pose of keyframe *x*_1_ is ***T***_**1**_, and the pose of keyframe *x*_2_ is ***T***_**2**_, the relative poses of these two keyframes are:

(13) $$T_{21}=T_{1}*T_{2}^{-1}$$

***T***_**21**_ is the estimation item, which is directly calculated from the camera pose. IMU pre-integration is equivalent to a measure between keyframes. Then the error term can be obtained:

(14) $$r_{21}=T_{1}*T_{imu}^{-1}$$

The keyframe pose is optimized by nonlinear optimization of the error term function in [30]. The residual term of the IMU can be expressed as:

(15) $$r_{B}\left(z_{b_{k}b_{k+1}},\chi \right)=\left[\begin{array}{c} \begin{array}{c} r_{p}\\ r_{q}\\ r_{v} \end{array}\\ r_{b_{a}}\\ r_{b_{g}} \end{array}\right]=\left[\begin{array}{c} q_{\omega b_{i}}^{*}(p_{\omega b_{j}}-p_{\omega b_{i}}-v_{i}^{\omega }∆t+\frac{1}{2}g^{\omega }∆t^{2}+)-\alpha_{b_{i}b_{j}}\\ 2{\left[q_{b_{i}b_{j}}^{*}⊗\left(q_{\omega b_{i}}^{*}⊗q_{\omega b_{i}}\right)\right]}_{xyz}\\ q_{\omega b_{i}}^{*}\left(v_{j}^{\omega }-v_{i}^{\omega }+g^{\omega }△t\right)-\beta_{b_{i}b_{j}}\\ b_{j}^{a}-b_{i}^{a}\\ b_{j}^{g}-b_{i}^{g} \end{array}\right]$$

#### 2.3.3. Graph Optimization Model

Compared with the loosely coupled method, the tightly coupled method can obtain higher trajectory accuracy [10,11]. The state variables in the tightly coupled approach will become continually larger in size with the operation of the algorithm. In order to limit the computational cost, a graph optimization method based on sliding windows [31] is adopted. The state quantity to be optimized in the sliding window is:

(16) $$\chi =\left[x_{1},x_{2},x_{3},\ldots ,x_{N},\lambda_{1},\lambda_{2},\lambda_{3},\ldots ,\lambda_{M},O_{1},,O_{2},O_{3},\ldots ,O_{O},T_{c}^{b}\right]$$

(17) $$x_{k}={\left[p_{wb_{k}},q_{wb_{k}},v_{k}^{w},b_{a}^{b_{k}},b_{g}^{b_{k}}\right]}^{T},k\in \left[1,N\right]$$

*x*_*k*_ is the status of the camera in the sliding window; *p*_*wbk*_ is the position of the camera; *q*_*wbk*_ is the orientation of the camera; $v_{k}^{w}$ is the speed of the camera; $b_{a}^{b_{k}}$ is the bias of the accelerometer; $b_{g}^{b_{k}}$ is the bias of the gyroscope; λ_*k*_ is the inverse depth of the 3D point; O_*k*_ is the orthogonal expression of the feature line; $T_{c}^{b}$ is the external parameter from the camera to the IMU; *N* is the number of keyframes in the sliding window; *M* and *O* are the number of point marks and line marks observed in all keyframes in the sliding window, respectively. As the algorithm runs, the oldest keyframe state variables in the sliding window need to be removed, and new keyframe state variables need to be added. The culled oldest frame state variables contain a lot of prior information.

The algorithm for graph optimization based on sliding windows not only uses the IMU data for state prediction but also IMU data as measurement information to optimize *X*. The optimal estimate of state *X* can be obtained by minimizing the residual of the state quantity within the sliding window, and its specific form is:

(18) $$\begin{array}{ccc} \chi_{MLE}=argmin & ({\|r_{p}-J_{p}\chi \|}^{2}+\underset{\left(i,j\right)\epsilon K}{\sum }\rho \left({\|r_{f}({\hat{Z}}_{f_{k}}^{C_{i}},\chi )\|}^{2}\right)+\underset{k\epsilon P}{\sum }\rho \left({\|r_{B}\left(z_{b_{k}b_{k+1}},\chi \right)\|}^{2}\right) & \\ & +\underset{\left(i,j\right)\epsilon K}{\sum }\rho \left({\|r_{L}\left(z_{L_{j}}^{c_{k}},\chi \right)\|}^{2}\right)) \end{array}$$

**Figure 5:**
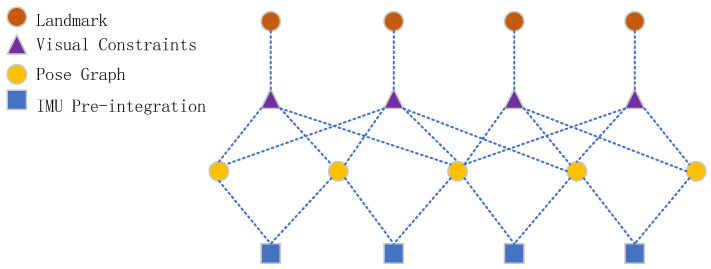
Data association.

*ρ* (∙) is a robust kernel function to suppress false matching of outliers; *r*_*p*_ is the prior residual; *r*_*c*_ represents the reprojection residual of visual point features; *r*_*L*_ represents the reprojected residual of visual line features; *r*_*B*_ represents the reprojection residual of the visual line features of the IMU pre-integration residuals; $z_{L_{j}}^{c_{k}}$ is the observation at time *k* relative to road sign *j*; $z_{b_{k}b_{k+1}}$ is the observed value of pose transformation obtained through IMU data at time *k* and time *k* + 1. As shown in Figure 5, in order to optimize the constraints between variables, the points and lines in the image that have a co-view relationship are associated with poses through visual constraints. Motion information between image keyframes is associated with IMU pre-integration constraints.

### 2.4. Closed-Loop Detection and Global Pose Optimization

The system error is effectively constrained by the optimization based on the sliding window, but the accumulated error still exists. For the accumulated error, the closed-loop detection is used to determine whether the historical position has ever been reached. If successful detection of a return to the historical position is achieved, a closed-loop constraint is formed. When sufficient closed-loop constraints are obtained, they can be used for the optimization of global poses to reduce the cumulative error.

The detection of closed-loop in visual SLAM is to find image similarity. A certain class of vectors with similar features in an image is called a word, and a dictionary is a collection of visual words. The closed-loop detection based on the Bag of Words (BoW) model [32] is effective. The feature points in the image are described using the Bag of Words model to determine which word the feature points belong to, and the set of words of different classes of feature points in the image constitutes a dictionary. The feature points in the current key frame image and the dictionary elements are compared by the bag of words to determine whether the images are similar and whether closed-loop is detected. The candidate frame with the highest similarity to the current frame is detected; feature matching and geometric verification is performed; and when the matching feature points reach a sufficient number, it is considered to constitute a closed loop and global bit pose optimization is performed, otherwise, the image words are added to the dictionary.

**Figure 6:**
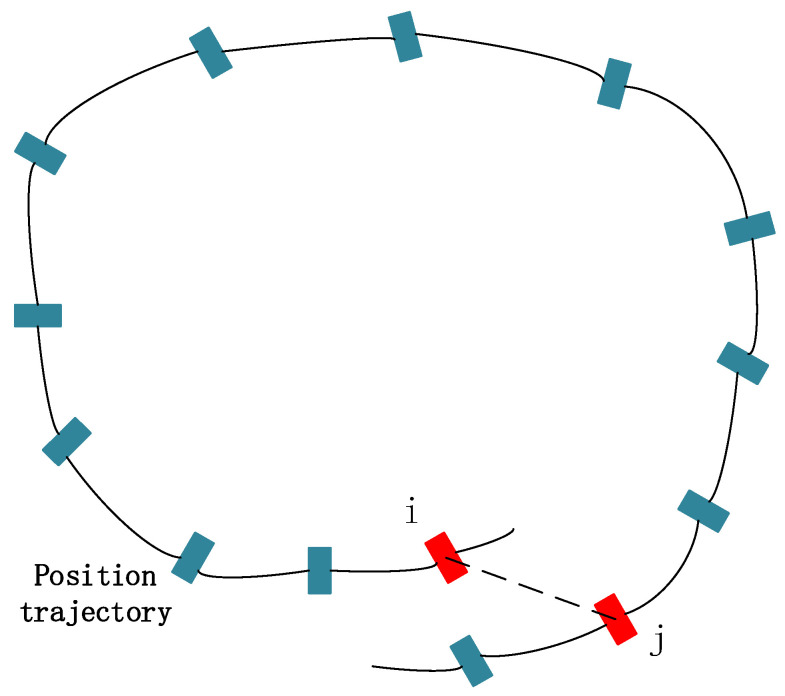
Closed-loop detection.

The global pose optimization is to perform pose adjustment when a closed-loop is detected. As shown in Figure 6, the pose of the current frame is *T*_*j*_, and it is detected that the *i* frame in the history frame is a closed-loop pose of *T*_*i*_. The relative pose of the *i* frame and the *j* frame is *T*_*ij*_. Without accumulated error, *T*_*ij*_ is strictly equal to ${T_{i}}^{-1}T_{j}$, but due to accumulated error, the residual can be constructed as shown in Equation (19). Adjust the pose to minimize the residual term to eliminate accumulated errors.

(19) $$e_{ij}=ln{\left({T_{ij}}^{-1}{T_{i}}^{-1}T_{j}\right)}^{\vee }$$

## 3. Results and Discussion

The EuRoC dataset was used to simulate the front-end vision processing part and the SLAM algorithm based on a monocular camera and IMU fusion respectively. It was obtained by using drone-mounted image sensors and IMU to collect data from 30 m^2^ ordinary rooms and 300 m^2^ factory environments. The dataset provided binocular camera images at 20 Hz and IMU measurements at 200 Hz. The pose transformation and trajectory of the aircraft during motion were obtained through the millimeter-level motion tracking system, and were used as the true values for the verification of the algorithm below.

In the indoor scene experiment, the algorithm in this paper was deployed on the Manifold-2C computing platform [33], which combined image data collected by visual sensors and IMU data to perform pose calculations in the laboratory scene.

Images with drastic changes in illumination, sparse texture, and poor illumination in the sequence were selected to test the visual front-end. Feature points and feature lines were extracted to produce accuracy statistics. The test for the overall positioning ability of the algorithm was to use the algorithm to estimate the poses of eleven sequences and to compare and evaluate these with the true value. Finally, the results were analyzed to verify the reliability and validity of our improved algorithm under different conditions.

### 3.1. Feature-Matching Evaluation

In the V1_03_difficult sequence, there were image frames with dramatic lighting changes and few textures. Two consecutive frames with large exposure differences in the sequence were selected to evaluate the robustness of the front-end feature extraction and matching algorithm.

**Figure 7:**
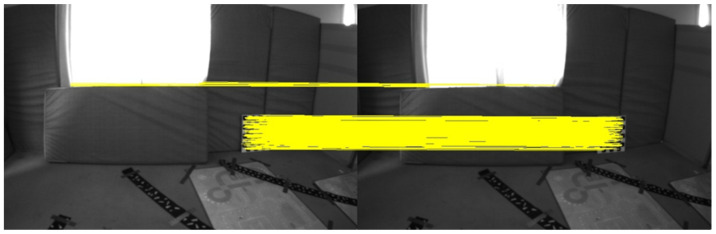
Results of feature point extraction and matching.

**Figure 8:**
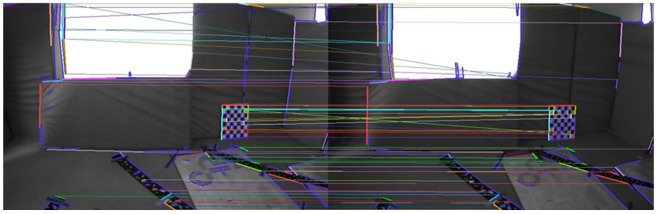
Results of feature line extraction and matching.

The feature points and feature lines of the two sequences of images were extracted and matched. The results are shown in Figure 7 and Figure 8.

**Figure 9:**
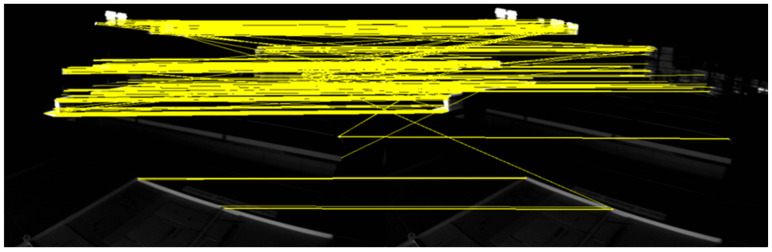
Image with insufficient illumination.

**Figure 10:**
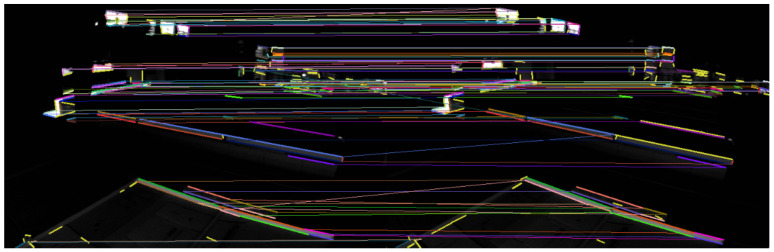
Image with insufficient illumination.

There are darker image frames with insufficient lighting in the MH_05_difficult sequence. The feature points and feature lines of the two sequences of images, respectively, were extracted and matched. The results are shown in Figure 9 and Figure 10.

The image frames selected in the V1_03_difficult sequence had drastic changes in illumination and sparse texture, and the matching pairs of feature points were mainly concentrated in the regions with obvious features. The image frames selected in the MH_05_difficult sequence were due to insufficient lighting, and the matching pairs of feature points were concentrated in local areas and had obvious mismatches. The matching pairs of feature lines were evenly distributed in the scenes with drastic changes in illumination and sparse textures, or scenes with insufficient lighting and relatively dark scenes. It can be seen that the geometric line feature had better robustness in the case of poor lighting. The matching accuracy rates of different features are shown in Table 1.

The correct rate of feature-point matching is significantly lower than that of feature line matching. The extraction and matching of feature lines have better robustness to scenes with changing illumination. Using feature points combined with feature lines in the visual front-end can effectively improve the robustness in scenes with severe illumination changes and avoid feature loss.

### 3.2. Odometer Accuracy Evaluation

**Figure 11:**
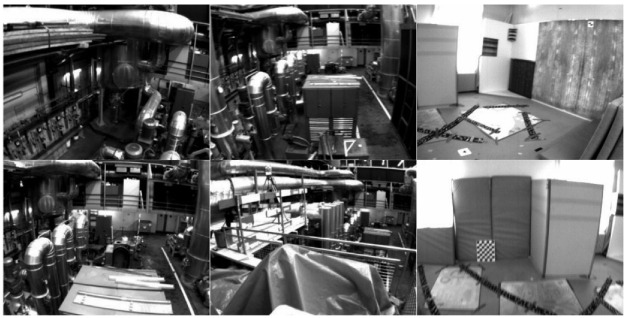
Partial dataset image.

Taking the MH_02 data sequence as an example, a total of 3040 sets of image information and 30,400 IMU information were provided. This algorithm used a monocular camera and selected the image information of cam0 as the input image data. The size of the image was an 8-bit grayscale image of 752 × 480 pixels. Some input images are shown in Figure 11. The IMU data formats in the dataset are timestamp, 3D angular velocity vector, and 3D acceleration vector. The final evaluation standard adopts the absolute pose error (APE) and compares it with the VINS-Mono algorithm.

**Figure 12:**
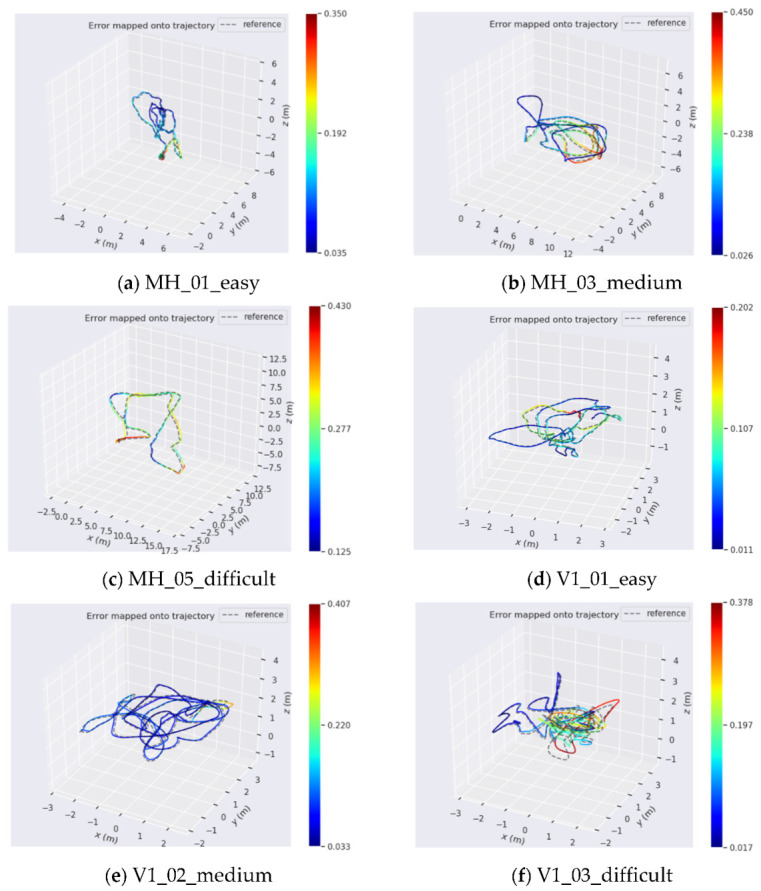
Comparison of trajectory error.

Figure 12 shows the trajectory error results of the 6 sequence ground-truth trajectories (dotted lines) in the EuRoc dataset and the trajectories (solid lines) obtained by our algorithm.

The absolute trajectory error APE is represented as a color. The trajectory calculated in this paper was relatively close to the true value trajectory in the dataset, but the cumulative error increased with the distance. From the trajectory calculation effect of each sub-sequence, the cumulative error was smaller in the sequence with good texture, good lighting, slow movement, and good lighting (the red trajectory is less). It showed that motion and lighting conditions have a great influence on the accuracy of the algorithm. APE was used to quantitatively analyze the pose error obtained by the algorithm in this paper, and to compare it with the trajectory error obtained by the VINS-Mono algorithm, as shown in Table 2.

The visual IMU fusion positioning and mapping algorithm designed in this paper were deployed on the Manifold-2C computing platform, which was configured with Intel Core i7-8550U; 8 GB 64 bit DDR4 2400 MHz RAM; 256 GB SSD, using Intel’s RealSense D435i camera as the sensor. The D435i output maximum resolution was 1920 × 1080 p, 30 fps. The D435i integrated an IMU model BMI055, and the output frequency of the IMU was 200 Hz. The camera and IMU were calibrated by Kalibr and imu_utils tools, and the relevant parameters of the calibration are shown in Table 3.

**Figure 13:**
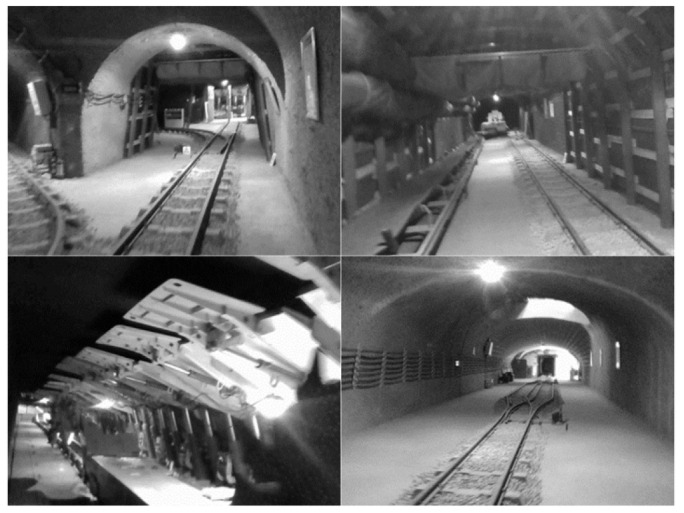
Scenes from coal mine tunnel.

Compared with the small scene in the indoor laboratory, the coal mine tunnel scene has poor lighting and sparse features, which can better test the robustness of the algorithm. In order to evaluate the performance of the fusion algorithm in special scenarios, tests were carried out in coal mine tunnels. The experimental environment was a simulated tunnel of a coal mine in the school, and the scene is shown in Figure 13.

**Figure 14:**
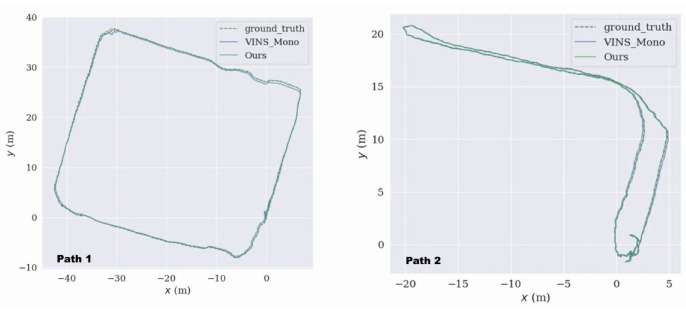
Trajectory error between the two algorithms and the true value under two paths.

Because there was no motion capture device to obtain the trajectory of motion in the coal tunnel, we used the laser SLAM results as the true value. We used the EVO tool to plot the trajectories of the proposed method in this paper and VINS_Mono under two paths as shown in Figure 14. Where the dashed line represents the true value of the trajectory, the blue line represents the trajectory calculated by VINS_Mono, and the improved method in this paper is represented by the green line. Table 4 shows the APE between the trajectories and the true values.

The above table shows the maximum deviation, average deviation, minimum deviation, RMSE, and standard deviation of the trajectories estimated on the two paths, respectively, for each of the two different algorithms for the relative translations. The best results are shown in bold. Numerically, the proposed method achieved the RMSE of the estimated tracking trajectory as low as 0.231 m and 0.052 m for both paths, respectively, which outperforms the comparative algorithms of 0.37 m and 0.129 m. It indicates that the proposed method in this paper achieves a higher localization accuracy under both coal mine tunnel paths.

## 4. Conclusions

In this paper, we propose a localization and mapping method for coal mine tunnel localization incorporating visual features and IMU, which uses data from IMU to assist monocular cameras for motion estimation. We discarded optical flow tracking and used ORB features and line features for feature matching to reduce feature loss due to fast motion. IMU provided motion compensation to reduce cumulative error. The robustness of the visual-inertial odometer in environments with poor lighting conditions was increased by closely coupling the IMU with the image features. Finally, by introducing closed-loop detection, visual information was fully utilized to obtain more accurate sensor motion trajectories. Experiments were conducted on the EuRoc dataset to compare the trajectories of the algorithm proposed in this paper with the VINS-Mono algorithm. The actual environment is verified in a simulated coal mine tunnel. The results showed that the trajectories obtained by the algorithm proposed in this paper were more accurate and more robust in the scenarios of a coal mine tunnel.

## Figures and Tables

**Table 1:** Result of feature extraction and matching.

Feature Type	V1_03_Difficult Correct Rate	V1_05_Difficult Correct Rate
Point	79.4%	68.6%
Line	**86.7%**	**89.7%**

**Table 2:** APE root mean square error(RMSE) in EuRoc datasets.

Sequence	Track Length	Test Conditions	RMSE	
			VINS-Mono	Ours
MH_01_easy	80.6 m	Situation A ^1^	0.137 m	**0.122 m**
MH_02_easy	73.5 m	Situation A	0.143 m	**0.134 m**
MH_03_medium	130.9 m	Situation B ^2^	2.263 m	**0.155 m**
MH_04_difficult	91.7 m	Situation C	0.362 m	**0.347 m**
MH_05_difficult	97.6 m	Situation C ^3^	0.377 m	**0.302 m**
V1_01_easy	58.6 m	Situation D ^4^	**0.080 m**	0.087 m
V1_02_medium	75.9 m	Situation B	0.201 m	**0.110 m**
V1_03_difficult	79.0 m	Situation C	0.201 m	**0.187 m**
V2_01_easy	36.5 m	Situation A	0.088 m	**0.086 m**
V2_02_medium	83.2 m	Situation B	0.158 m	**0.148 m**
V2_03_difficult	86.1 m	Situation C	0.307 m	**0.277 m**

^1^ Test equipment moving slowly in good light. ^2^ Test equipment moving fast in good light. ^3^ Test equipment moving fast in poor light. ^4^ Test equipment moving slowly in poor light.

**Table 3:** Parameters of the camera and IMU.

Item	Parameters
Camera internal parameters	$\left[\begin{array}{ccc} 607.78 & 0 & 321.35\\ 0 & 608.07 & 236.30\\ 0 & 0 & 1 \end{array}\right]$
Camera distortion parameters $\left[\begin{array}{cc} \begin{array}{cc} k_{1} & k_{2} \end{array} & \begin{array}{cc} p_{1} & p_{2} \end{array} \end{array}\right]$	$\left[\begin{array}{cc} \begin{array}{cc} 0.0765 & 0.0185 \end{array} & \begin{array}{cc} 0.00006387 & 0.00003974 \end{array} \end{array}\right]$
Accelerometer noise	0.0187 m/s^2^
Accelerometer Random Walk	0.000596 m/s^2^
Gyroscope noise	0.0018 rad/s
Angular Random Walk	0.000011 rad/s

**Table 4:** Error statistics between the two algorithms and the true value under two paths.

APE	Path1		Path2	
	VINS_Mono	Ours	VINS_Mono	Ours
Max	0.879 m	**0.660 m**	0.219 m	**0.207 m**
Mean	0.330 m	**0.192 m**	0.120 m	**0.046 m**
Min	0.028 m	**0.005 m**	0.025 m	**0.004 m**
RMSE	0.370 m	**0.231 m**	0.129 m	**0.052 m**
Std	0.167 m	**0.130 m**	0.047 m	**0.026 m**

## Data Availability

Not applicable.
